# Mobile Personal Health Monitoring for Automated Classification of Electrocardiogram Signals in Elderly

**DOI:** 10.1155/2018/9128054

**Published:** 2018-05-29

**Authors:** Luis J. Mena, Vanessa G. Félix, Alberto Ochoa, Rodolfo Ostos, Eduardo González, Javier Aspuru, Pablo Velarde, Gladys E. Maestre

**Affiliations:** ^1^Academic Unit of Computing, Master Program in Applied Sciences, Universidad Politecnica de Sinaloa, Mazatlan 82199, Mexico; ^2^Department of Electronic, Faculty of Mechanical and Electrical Engineering, Universidad de Colima, Colima 28400, Mexico; ^3^Academic Program of Electronic Engineering, Universidad Autonoma de Nayarit, Tepic 63000, Mexico; ^4^Department of Biomedical Sciences, Division of Neurosciences and Department of Human Genetics, University of Texas Rio Grande Valley School of Medicine, Brownsville 78520, USA

## Abstract

Mobile electrocardiogram (ECG) monitoring is an emerging area that has received increasing attention in recent years, but still real-life validation for elderly residing in low and middle-income countries is scarce. We developed a wearable ECG monitor that is integrated with a self-designed wireless sensor for ECG signal acquisition. It is used with a native purposely designed smartphone application, based on machine learning techniques, for automated classification of captured ECG beats from aged people. When tested on 100 older adults, the monitoring system discriminated normal and abnormal ECG signals with a high degree of accuracy (97%), sensitivity (100%), and specificity (96.6%). With further verification, the system could be useful for detecting cardiac abnormalities in the home environment and contribute to prevention, early diagnosis, and effective treatment of cardiovascular diseases, while keeping costs down and increasing access to healthcare services for older persons.

## 1. Introduction

Cardiovascular diseases (CVD) have remained the leading cause of death globally during the last 15 years. An estimated 17.7 million people died from CVD in 2015, representing 31% of all global mortality. Of these deaths, approximately 6.9 million were in people aged 60 years and older, and over 75% occurred in low and middle-income countries (LMIC) [1, 2]. LMIC are more greatly affected than high-income countries [3–5], largely because people with low socioeconomic status have poor access to healthcare for early diagnosis and treatment of CVD [5]. An increasing urgency exists to tackle CVD in LMIC through effective strategies, guided and monitored by robust estimates of disease prevalence and burden [6]. Thus, technological innovations, including mobile and wireless technologies, are now being developed to improve prevention and control of CVD, and other aspects of healthcare, particularly for older people residing in LMIC [7–9].

The growing application of smartphone technology, due to decreasing costs and increased ease-of-use, combined with parallel advances in sensing technologies, is causing a shift from traditional clinic-based healthcare to real-time monitoring. This shift is supported by the development of mobile personal health monitor (PHM) systems, which are personalized, intelligent, reliable, and noninvasive [10, 11]. PHM systems could improve the quality of care, while reducing costs through timely detection [12–14].

**Figure 1:**
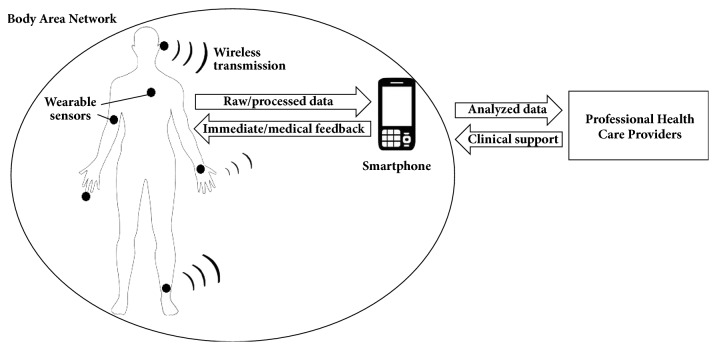
General architecture of a mobile personal health monitor system.

Mobile PHM systems typically consist of a Body Area Network—a set of wearable sensors with wireless data transfer and energy storage capability—integrated by a smartphone as the central processing unit (Figure 1). The physiological signals are processed in real-time by applying machine learning techniques, providing immediate feedback to the user. The data can also be made available to healthcare providers for medical feedback and clinical support [15–17].

PHM systems that offer mobile electrocardiogram (ECG) monitoring have received increasing attention in recent years [18–20]. ECG records are used for screening, diagnosis, and monitoring of several heart conditions from minor to life threatening. Hence, ECG monitoring is a critical and an essential part of healthcare delivery for older adults [17]. Therefore, PHM systems that incorporate ECG data would offer mobile physiological, diagnostic, prognostic, therapeutic, surveillance, and archival capabilities [18, 19] in a wide range of situations, including rural zones, areas lacking cardiologists, and population of solitary elderly, many of whom live alone in their own homes and are restricted physically [21].

However, although a number of PHM systems that collect ECG data have been developed, some of these do not include classification methods for automated detection of arrhythmias or other abnormalities. Among those validated, Kwon et al. proposed a smartphone-integrated ECG monitoring system that works opportunistically during natural smartphone use [22]. The system captured ECG reliably in target situations with a reasonable rate of data drop. Depari et al. developed a single-lead ECG tracing acquisition system based on a smartphone, with a purposely designed application to demodulate the audio signal and extract, plot, and store the ECG tracing [23]. Dinh designed a wearable unit for detecting and sending ECG signals wirelessly to a smartphone [24]. Yu et al. developed a wireless two-lead ECG sensor that transmitted data via Bluetooth and processed and displayed the ECG waveform on a smartphone, all with low power consumption for long-term monitoring [25].

Other PHM systems use commercial monitors or do not provide an intrinsic method for classify ECG signals. Lee et al. designed a wireless system for acquisition and classification of ECG beats integrated with a smartphone. Abnormal beats and other symptoms were diagnosed by cardiologists from results displayed on the screen. Accuracy of beat classification was 97.25% [26]. Miao et al. developed a wearable ECG monitoring system using a smartphone, with automated recognition of abnormal patterns via decision trees in a WEKA environment [27]. The system achieved a 2.6% discrimination ability [28]. Oresko et al. developed a smartphone-based application for real-time CVD detection, using a commercial ECG heart monitor and an adaptive artificial neural network (NN) algorithm for signal preprocessing and classification [29]. The system was trained using the MIT-BIH arrhythmia database [30] and retrained based on real ECG recordings, ultimately demonstrating classification accuracy of 93.32%. None of the aforementioned studies [22–26, 28, 29] reported considerations in software design to address end-user usability and acceptance of mobile PHM systems in older adults.

To improve on previous systems, it would be necessary to enhance the capture as well as the automated classification of ECG signals. We developed a complete mobile PHM system, integrated with a self-designed wireless sensor for ECG signal acquisition, and a native purposely designed smartphone application to be user-friendly to elderly, based on machine learning techniques, for automated classification of captured ECG beats. The signal sensing and transferring process uses a two-lead ECG sensor with Bluetooth technology and an artificial NN approach for identifying abnormal ECG patterns.

The rest of this paper is organized as follows. The methodology of the proposed PHM system is presented in detail in Section 2; the experimental results for ECG signals acquisition, wireless transmission, and assessment of recognition accuracy are shown in Section 3; we conclude our study in Sections 4 and 5, with discussion, limitations, and perspective for further research.

## 2. Materials and Methods

**Figure 2:**
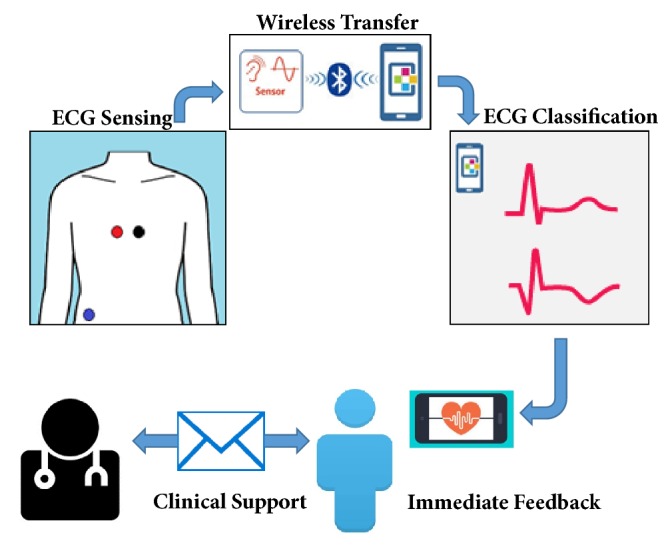
Framework of the mobile personal health monitor system.

The PHM system described in this report operates in five stages: sensing, transferring, classification, immediate feedback, and clinical support (Figure 2). The captured ECG tracings are transmitted and displayed in real-time on a smartphone screen. The presence or absence of arrhythmias, determined using machine learning analysis, is included and is shared via email with healthcare professionals for verification of abnormal ECG patterns.

### 2.1. ECG Sensor

The sensor design includes acquisition, amplification, filtering, digitalization, and transmission of ECG signals. Three identically sized electrodes and low frequency amplifiers are used to capture the signals and the coupling of impedances. The signal is filtered through low-pass and high-pass filters to improve the signal/noise ratio. The processed signal is then digitized and transmitted by an analog-to-digital converter and Bluetooth module embedded in a microcontroller unit. A 9V primary lithium battery with 1200 mAh capacity powers the ECG sensor.

**Figure 3:**
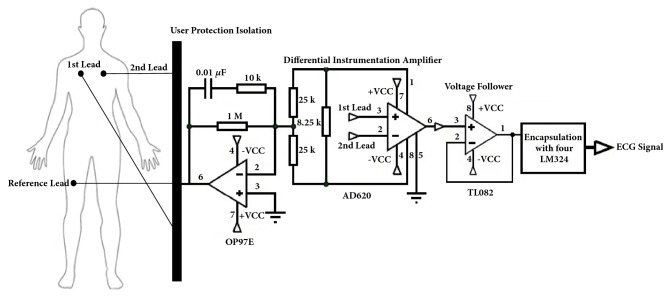
Schematic representation of the ECG amplifier circuit and electrode placement on the body.

To acquire reliable ECG signals, two electrodes are attached to the chest as precordial leads V1 and V2 positioned in the fourth intercostal space to the right and left of the sternum, respectively, because incorrect positioning of the precordial electrodes changes the ECG significantly [31], and a reference electrode is placed far from these on the right leg (Figure 3). The reference electrode plays the role of driving the user's body to attenuate the common mode noise caused by external electromagnetic interference [32]. The analog input signal from two lead electrodes was initially amplified through an AD620 differential instrumentation amplifier [33]. Before the next amplifier stage, we coupled the impedance using a TL082 operational amplifier, configured as a voltage follower [34].

**Figure 4:**
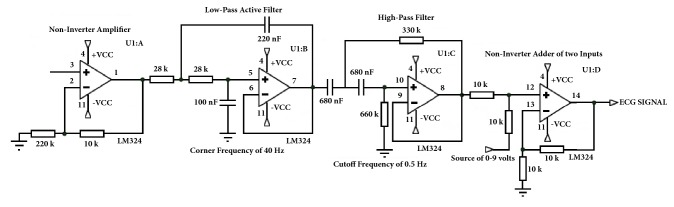
Encapsulation with four LM324 operational amplifiers to amplify, filter, and add voltage to the ECG signal.

The current configuration uses an instrumental amplifier, based on an encapsulation with four LM324 operational amplifiers [35], to amplify the signal with a noninverter amplifier, then filter it, and add voltage (Figure 4). A low power OP97E operational amplifier [36] closes the circuit, protects the user from static charges, and suppresses voltage transients. Two LM324 operational amplifiers act as Butterworth filters, to generate an appropriate low-noise signal that fits within the input range of the analog-to-digital converter [37]. A low-pass active filter with a corner frequency of 40 Hz and a second-order high-pass filter with cutoff frequency of 0.5 Hz remove unnecessary frequency components of the ECG signal. Because the signal obtained consists of positive and negative parts, it was necessary to add a positive carrier signal. To recompose the signal, we used operational LM324 amplifiers as noninverter adders of the two inputs, fed by the ECG signal and a variable power source of 0–9 volts. This increases or decreases the carrier signal, as appropriate. A pair of equal resistances is added, one to the input of the analog signals and another from the inverter input of the operational amplifier to the circuit ground. Thus, the output signal has the same frequency, but with only positive voltage values, and is ready to be read by any microcontroller.

The Blend Micro of Read Bear Labs [38], which combines the Atmega32U4 microcontroller unit with a Bluetooth Low Energy (BLE) module [39, 40], is used for microcontroller processing of the signal. Generic Access Profile (GAP) controls connections and advertising in BLE standard and determines how two devices interact with each other by assigning roles. The ECG sensor and smartphone are defined as peripheral and central devices, respectively. GAP sends advertising out as Advertising Data payload, which can contain up to 31 bytes of data and constantly transmits from the sensor to the smartphone. After a dedicated connection is established, the advertising process stops, and BLE uses Generic Attribute Profile (GATT) services and characteristics to communicate in both directions. This connection is exclusive, because a BLE peripheral only can be connected to one central device at a time.

Communication is established through a generic data protocol, Attribute Protocol, which is used to store services, characteristics, and related data in a simple lookup table. GATT transactions in BLE operate as a server/client relationship. The GATT server is the peripheral that holds the Attribute Protocol, and the GATT client (smartphone) sends requests to this server. All transactions are started by the master device, the smartphone, which receives responses from the slave device, the ECG sensor. A simple Universal Asynchronous Receiver Transmitter type interface [41] defines a custom service containing two specific characteristics for the channels of transmission and reception of the ECG signal.

### 2.2. Neural Network Approach

We use a three-layered, feedforward NN approach, built through Matlab NN toolbox [42], for automated classification of acquired ECG tracings. A scaled conjugate gradient back-propagation algorithm with random weights/bias initialization is used for the training stage. The transfer functions are sigmoidal hyperbolic, logarithmic tangential, and lineal. Performance of the NN system was tested with a cross-entropy error function using the mean-squared error parameter, computed for differences between the actual outputs and the outputs obtained in each trained step. The training ended if the total sum of the squared errors was <0.01 or when 3000 epochs were reached. The target outputs for normal and abnormal ECG patterns were (0,1) and (1,0), respectively.

### 2.3. Data Processing

ECG data for training was obtained from a publicly available source, the Physikalisch-Technische Bundesanstalt Diagnostic ECG Database [43]. This benchmark database contains 549 two-minute digitized ECG records of 290 subjects (mean age 57.2 y; 27.9% women) provided by the National Metrology Institute of Germany. The ECG data includes 15 simultaneously measured signals: the conventional 12 leads, plus 3 Frank Lead ECGs. Each signal is digitized at 1000 samples per second, with 16-bit resolution over a range of ±16 mV and 1 KHz sampling frequency.

We selected data from 268 subjects with clinical summaries available. These included a variety of diagnostic classes: 52 healthy controls, 148 myocardial infarctions, and 68 with other cardiac abnormalities. ECG beats were classified in normal and abnormal heartbeat patterns from ECG records reported as regular and irregular cardiac rhythm. Lead V1 was chosen for the analysis, because it has the highest ratio of atrial to ventricular signal amplitude and, therefore, offers more representative characteristics for identifying the common heart diseases [44, 45]. To avoid overfitting and improve the generalization capability of the NN approach, we added simulated ECG data with artificial corruption, using a Gaussian white-noise model [46], to generate 110 normal and 72 abnormal virtual ECG tracings. The global training dataset contained 8000 beats from all 450 records, for feature extraction of ECG patterns.

The trained NN system was tested on participants of the Maracaibo Aging Study [47], which has 2500 subjects ≥ 55 y of age. One hundred voluntary subjects (mean age 73.5 ± 11.8 y; 74% women) were recruited in the Institute for Biological Research of the University of Zulia, in Maracaibo, Venezuela. All 100 subjects had a previous ECG diagnostic performed by an expert cardiologist, and 13 were diagnosed with some type of cardiac arrhythmia. These ECG records were classified as abnormal and the rest as normal ECG patterns. Recruited participants had reasonable smartphone skills and were assertive about using new technologies. Each volunteer was instructed how to use the smartphone application and underwent 16-second ECG monitoring using the PHM system. ECG acquisitions were performed and supervised by medical staff. The ethical review board of the Institute of Cardiovascular Diseases of the University of Zulia approved the protocol. Informed consent was obtained from each subject or a close family member.

### 2.4. Software Development

We use Matlab Compiler SDK to save the trained NN as a Matlab function into a shared library for use in an external framework [48]. The smartphone application for plotting the acquired ECG tracing on screen and for return NN output was developed in an Android Studio development environment. The Android Bluetooth serial port profile library [49] establishes the connection with the wearable ECG sensor. Android multithreading [50] allows the smartphone to maintain normal operations, while receiving real-time ECG signals. To make the Android application user-friendly to elderly subjects with reduced vision and manual dexterity, we use a simplified Graphic User Interface with a bright screen, large text and numbers, and simple input buttons with touchscreen technology, all of which have been proven to be efficient for older adults [51]. To provide accurate diagnostic and medical support, application settings include the option to sending screenshots of ECG signal and classification results via email to previously specified healthcare professionals. To assure privacy, reports forwarded to selected recipients lack personal identification, which is already associated with the source email address. The system can be configured to automatically send ECG profiles at the end of each monitoring period or only when abnormal ECG patterns are detected.

## 3. Results

### 3.1. Acquisition of ECG Signal

**Figure 5:**
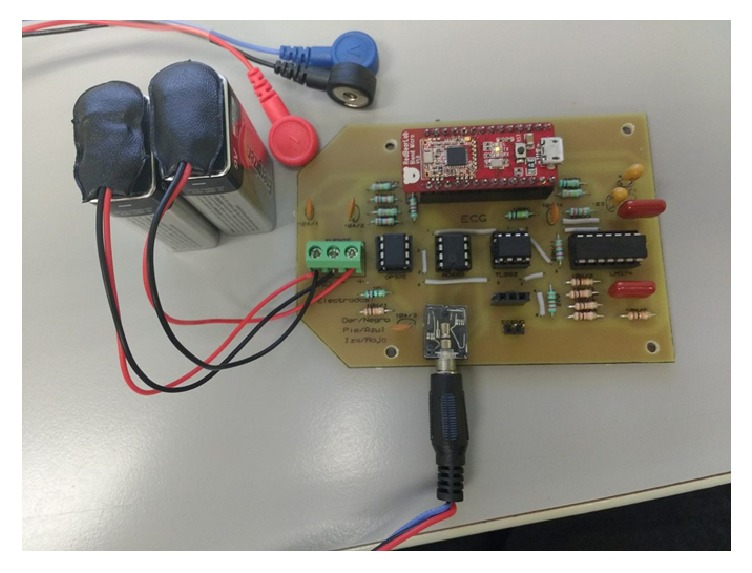
Prototype of the self-designed ECG sensor device.

**Figure 6:**
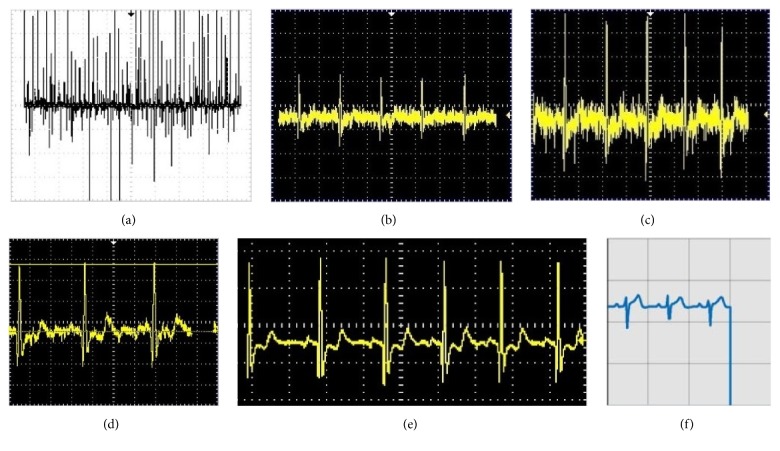
ECG signal processing: (a) first stage of amplification; (b) impedance coupling; (c) second stage of amplification; (d) low-pass filtering; (e) high-pass filtering; (f) positive ECG signal on smartphone screen.

**Figure 7:**
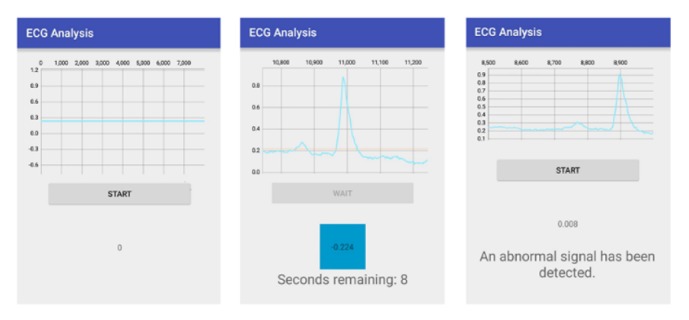
Screenshots of ECG analysis process on smartphone.

A prototype of the PHM system is shown in Figure 5, and the performance characteristics of the ECG sensor device are given in Table 1. Processing of the ECG tracing, from the first stage of amplification to display on the smartphone, includes (a) amplification by the AD620, (b) coupling of impedance through the TL082, (c) amplification through the LM324, (d) filtering through the low-pass filter, (e) filtering with the high-pass filter, and (f) digitalization and transmission of the positive ECG signal (Figure 6). The analytical process is displayed on the smartphone (Figure 7).

### 3.2. ECG Classification

**Figure 8:**
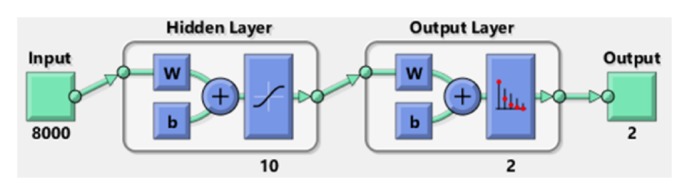
Neural network architecture with the best performance.

When the NN approach was trained on 450 records of the training dataset, the mean-squared error convergence goal (0.0052) was reached in 802 epochs. The best performance was obtained using 10 neurons in the hidden layer of the NN system (Figure 8). Overall classification accuracy in training stage was 97.3%. Correct classification was 92.6% for normal and 100% for abnormal ECG patterns.

When performance of the trained NN approach was tested on real ECG tracings from the test dataset, classification accuracy was 97%. The results are shown in a confusion matrix, where each cell contains the number of ECG records classified for the corresponding combination of estimated and true outputs for normal and abnormal ECG patterns (Table 2).

The total test performance was determined by evaluation metrics (Table 3): accuracy (ratio of the number of correctly classified ECG signals to the total number of ECG signals classified), sensitivity (rate of correctly classified abnormal ECG signals among all abnormal ECG signals), specificity (rate of correctly classified normal ECG signals among all normal ECG signals), and precision (rate of correctly classified abnormal ECG signals among all of detected abnormal ECG signals). These metrics are relevant to performance for medical diagnosis applications [52]. Finally, a posterior survey indicated that the majority of the participants found the smartphone application easy to use and considered the time spent learning how to use the mobile ECG monitoring system was reasonable.

## 4. Discussion

Recent technological advances in integration and miniaturization of physical sensors and increasing computing capability of smartphones have enabled the development of mobile PHM systems as a cost-effective strategy to support healthcare that is focused on the consumer, transparency, convenience, and prevention [53]. Clinical studies reported high sensitivity and specificity at detecting atrial fibrillation [54] and other cardiac abnormalities using wireless mobile ECG devices [55–58]. The ability to provide pervasive heart monitoring to anyone at any time, through natural interactions between smartphone and user, overcomes constraints of place, time, and character and provides personalized information in a transparent form. Users can configure mobile PHM systems to their individual needs and preferences, taking into account age, gender, and ethnicity. Immediate feedback alerts the user of abnormal conditions or abrupt changes in near real-time, potentially improving outcomes. As a final point, clinicians can receive automated updates, providing structured CVD management while minimizing clinical visits.

On the other hand, results of a 2014 consumer survey, performed by PricewaterhouseCoopers Health Research Institute, showed that almost half of respondents were ready to have an ECG device attached to their smartphone, with results wirelessly sent to their physician [59]. Latest evidence from LMIC suggests that mobile PHM systems can improve lifestyle behaviors and healthcare management related to CVD, particularly for aged people and frail users [60].

Elderly should be the primary target of mobile ECG monitoring systems for several reasons. Mainly, because the population aged 65 and older is projected to be about 83.7 million in 2050 [61], worldwide epidemic of chronic diseases is strongly linked to population aging, and the leading contributors to disease burden in older people are CVD [6]. Nevertheless, mobile PHM systems remain in its nascent stages related to behavioral health and older adults [9].

While research in PHM systems have demonstrated feasibility and effectiveness across a variety of populations and health problems, studies generally exclude older adults or do not report significant age differences in responses to the interventions [9]. A possible explanation is the persistence of stereotypes that older adults are afraid, reluctant, and incompetent to use modern technology. Besides, seniors who may believe themselves incapable of learning to use new technologies perpetuate many of these stereotypes [62–64]. Therefore, usability and acceptance of mobile PHM by older adults is not only based on their healthcare requirements, but also on their perspective of technology. Since cognitive performance commonly declines with age, minimizing the complexity of smartphone applications and user-interactions could be key to the adoption of mobile PHM systems by elderly users and should be considered in stages of design and development [65].

In this sense, we developed a mobile PHM system for ECG monitoring and automated classification of heartbeat patterns to identify potential arrhythmias in elderly. The system combines a wearable wireless sensor, mobile technology, and machine learning techniques. Software design included specific characteristics aimed to improve usability and acceptance of older persons. User interface to display and classify ECG signals was simplified at one dedicated button to minimize the amount of steps to be memorized (Figure 7). Additionally, security mechanisms such as user identification and password were omitted to access smartphone application.

Our system has a number of advantages over previously developed mobile PHM systems for monitoring ECG signals, which do not report software design concept to address the user acceptability and acceptance issue in elderly [22–26, 28, 29], do not include automated classification [22–25], operate with commercial sensors [29], or do not provide internal methods for classifying arrhythmias [26, 28]. The prototype detected normal and abnormal ECG patterns in a group of older adults residing in a LMIC with a high degree of accuracy (97%), sensitivity (100%), and specificity (96.6%). Thus, our mobile ECG monitoring approach could be useful for detecting cardiac abnormalities in the home environment and contribute to prevention, early diagnosis, and effective treatment of CVD, while keeping costs down and increasing access to healthcare services for older persons.

However, the ECG monitoring and classification system described herein has several potential limitations. First, our system and most other mobile ECG monitors record a single-channel ECG signal, which provides more limited information than 12-lead ECG devices. Nevertheless, a recent study found good correlation between smartphone ECG and 12-lead ECG data, before and after antiarrhythmic drug therapy [66]. Second, despite high overall recognition, the precision of the NN classifier is only 81.3%, although false positive signals would be recognized by physician evaluation. Third, the system provides timely detection of abnormal ECG patterns for further diagnosis by healthcare professionals but does not identify specific types of cardiac disorders. Finally, the system was tested using a relatively small sample (n = 100) at a single center and primarily included Venezuelan females; thus, the system performance characteristics might not be generalizable to other user populations. Therefore, further studies are necessary to extend use of mobile ECG monitoring to other geographically diverse elderly populations as well as provide a better characterization of heart rhythm abnormalities.

## 5. Conclusions

The mobile ECG monitoring system described in this report provides near real-time data and automated classification of ECG signals from older adults. The machine learning classifier discriminates between normal and abnormal cardiac rhythms with high accuracy. With further development and verification, the system could provide a cost-effective strategy for primary diagnosis of potential arrhythmias and improve preventive healthcare, particularly in population of solitary elderly.

## Figures and Tables

**Table 1:** Performance summary of the ECG sensor device.

**Technology**	Low-Power Microchip 8-bit AVR RISC-Based Microcontroller
**Supply Voltage**	3.3 V
**Input Impedance**	100 MΩ
**Frequency Response**	Range 0.1Hz and Internal 8MHz Calibrated Oscillator
**Common Mode Rejection Ratio**	>90dB
**Gain**	45
**Sampling Rate**	9.6KHz
**Data Bit-Width**	8 bits

**Table 2:** Confusion matrix for classification of the test dataset.

	**True output**	
**Estimated output**	**Normal**	**Abnormal**
**Normal**	84	0
**Abnormal**	3	13

**Table 3:** Total test performance of the mobile PHM system.

**Evaluation metrics**	**Values (**%**)**
**Sensitivity**	100
**Specificity**	96.6
**Accuracy**	97
**Precision**	81.3

## Data Availability

The data used to support the findings of this study are available from the corresponding author upon request.
